# 

**Published:** 1866-03

**Authors:** 


### CONTENTS.

### ORIGINAL CONTRIBUTIONS.

ARTICLE IX. Practical Remarks on Blood-Letting. By D. B. Trimble, M.D., Chicago, ---- 129

ART. X. Surgical Cases. By Geo. K. Amerman, Attending Surgeon to the Chicago City Hospital, ---- 141

ART. XI. Mortuary Statistics of Natchez, Miss., for the Year ending Dec. 31st, 1865. Compiled by J. Stebbins King, M.D., Surgeon in charge Mississippi State Hospital, Natchez, Miss.; late Recorder Board of Health, ---- 144

ART. XII. Report of the Prevelence of Diseases, &c., During the month of December, 1865.—Broncho-Pneumonia complicated with Diarrhœa.—Cerebro-Spinal Meningitis. Read to the Chicago Medical Society by N. S. Davis, M.D., &c., ---- 147

### SELECTIONS.

Clinical Lecture on the Study of Children's Diseases. Given in the Hospital for Sick Children. By Charles West, M.D., Physician to the Hospital, ---- 150

Report of the Committee appointed to Memorialize Congress in Regard to the Medical Department of the Army, ---- 158

The Amaurosis and Deafness of Smokers and Drinkers. By MM. Sichel and Triquet, ---- 164

Blepharitis Ciliaris and Phlyctenular Conjunctivitis. By E. Williams, M.D., Prof. of Ophthalmology and Aural Surgery, ---- 166

On Anæsthetics. By J. M. Carnochan, M.D., Surgeon in Chief to the State Emigrant's Hospital, New York, &c., ---- 173

Epidemic, or Army Itch. By L. C. Butler, M.D., of Essex, Vt., ---- 174

### EDITORIAL.

Medical College Instruction, ---- 178

Cook County Hospital, ---- 179

Chicago Medical College Annual Commencement, ---- 179

Chicago Medical Society, ---- 182

Death of Dr. I. P. Lynn, ---- 185

Chicago Medical Journal, ---- 186

American Medical Association, ---- 186

Increased Use of Stimulants in the London Hospitals, ---- 187

Vaccine Matter from the Cow, ---- 189

